# Utilization of structured emergency transport system in optimizing burn center referral: a case record audit

**DOI:** 10.1186/1865-1380-7-S1-P8

**Published:** 2014-07-25

**Authors:** Vanmathy Venkatapathy, Bhavani Muthukrishnan, Murugan Thalaippan

**Affiliations:** 1 Government Royapettah Hospital, attached to Kilpauk Medical College, Chennai, India

## Objectives

To conduct an audit on the referral patterns of burns in our burns center [Government Kilpauk Medical College] based on National Burn Center Referral Criteria. 

To study the role of the 108 ambulance services in optimizing such patient referrals.

## Methods

A case record audit was conducted at Government Kilpauk Medical College having two burn units; one at the Kilpauk medical college Hospital and the other at Government Royapettah Hospital. Emergency department registers, burn center registers and case sheets of patients with burns who attended the Emergency Department, from January 2013 to December 2013 were studied. The adherence to national burn center referral guidelines based on American Burn Association Referral Criteria was analysed in two groups; those brought by Emergency Medical Services (108 EMRI/GVK ambulance) and those who came by other means to the emergency department.

## Results

During the study period a total of 874 case histories were retrieved from the accident registers. 108 GVK-EMRI were involved in transporting 12.6%, of which 6% were from outside the state of Tamilnadu. The age of patients ranged from 1 to 75 years. 80.54% were in the age group between 15-45 years. Pediatric burns comprised 16.3% of burn admissions. The majority of paediatric patients suffered from scalds while most of the domestic causalities were due to flame injuries. Eighteen cases (2%) were brought dead, of which 83% were due to electrocution. 70.6% of cases were recorded as accidental burns, 5% were from those who tried to rescue burn victims. Most of them had injuries that did not require burn center care. 48.5% were directly admitted to burns ward while 49.4% were sent for either plastic surgeon opinion or general surgeon opinion.

## Limitations

1. This audit was done to study the referral pattern only without an insight into the outcomes of those who got admitted.

2. The treatment instituted at the emergency department itself was not studied.

3. Our study is a retrospective one so loss of data was inevitable.

## Conclusion

Strict adherence to the national referral guidelines for burn injuries may have a positive impact on patient outcome but at the same time increase workload in burn centers, that may have economic implications in resource allocation nationally.

